# A chromosome-level genome assembly of the European green toad (*Bufotes viridis*)

**DOI:** 10.1093/g3journal/jkaf002

**Published:** 2025-02-19

**Authors:** Patrik Rödin-Mörch, Ignas Bunikis, Eunkyoung Choi, Claudio Ciofi, Genevieve Diedericks, Maria Angela Diroma, Elísabet Einarsdóttir, Kristofer Försäter, Julia Heintz, Linnea Jonsäll, Henrik Lantz, Anssi Laurila, Henrique G Leitão, Mai-Britt Mosbech, Chiara Natali, Remi-André Olsen, Olga Vinnere Pettersson, Lucile Soler, Hannes Svardal, Estelle Proux-Wéra, Jacob Höglund

**Affiliations:** Department of Ecology and Genetics/Animal Ecology, Uppsala University, Norbyvägen 18D, Uppsala 752 36, Sweden; Science for Life Laboratory, Department of Immunology, Genetics and Pathology, Uppsala University, National Genomics Infrastructure hosted by SciLifeLab, Box 518, Uppsala 751 08, Sweden; Science for Life Laboratory, Department of Gene Technology, KTH-Royal Institute of Technology, Solna SE-171 21, Sweden; Department of Biology, University of Florence, Sesto Fiorentino (FI) 50019, Italy; Evolutionary Ecology Group, Department of Biology, University of Antwerp, Antwerp 2020, Belgium; Department of Biology, University of Florence, Sesto Fiorentino (FI) 50019, Italy; Science for Life Laboratory, Department of Gene Technology, KTH-Royal Institute of Technology, Solna SE-171 21, Sweden; Foundation Nordens Ark, Åby Säteri, Hunnebostrand SE-456 93, Sweden; Science for Life Laboratory, Department of Immunology, Genetics and Pathology, Uppsala University, National Genomics Infrastructure hosted by SciLifeLab, Box 518, Uppsala 751 08, Sweden; Science for Life Laboratory, Department of Immunology, Genetics and Pathology, Uppsala University, National Genomics Infrastructure hosted by SciLifeLab, Box 518, Uppsala 751 08, Sweden; Department of Medical Biochemistry and Microbiology, Uppsala University, NBIS—National Bioinformatics Infrastructure Sweden, Box 582, Uppsala 751 23, Sweden; Department of Cell and Molecular Biology, Uppsala University; NBIS—National Bioinformatics Infrastructure Sweden, Box 596, Uppsala 751 24, Sweden; Department of Ecology and Genetics/Animal Ecology, Uppsala University, Norbyvägen 18D, Uppsala 752 36, Sweden; Evolutionary Ecology Group, Department of Biology, University of Antwerp, Antwerp 2020, Belgium; Science for Life Laboratory, Department of Immunology, Genetics and Pathology, Uppsala University, National Genomics Infrastructure hosted by SciLifeLab, Box 518, Uppsala 751 08, Sweden; Department of Biology, University of Florence, Sesto Fiorentino (FI) 50019, Italy; Science for Life Laboratory, Department of Biochemistry and Biophysics, Stockholm University, Solna 171 65, Sweden; Science for Life Laboratory, Department of Immunology, Genetics and Pathology, Uppsala University, National Genomics Infrastructure hosted by SciLifeLab, Box 518, Uppsala 751 08, Sweden; Department of Medical Biochemistry and Microbiology, Uppsala University, NBIS—National Bioinformatics Infrastructure Sweden, Box 582, Uppsala 751 23, Sweden; Department of Cell and Molecular Biology, Uppsala University; NBIS—National Bioinformatics Infrastructure Sweden, Box 596, Uppsala 751 24, Sweden; Evolutionary Ecology Group, Department of Biology, University of Antwerp, Antwerp 2020, Belgium; Naturalis Biodiversity Center, Leiden 2333, Netherlands; Science for Life Laboratory, Department of Biochemistry and Biophysics, Stockholm University, Solna 171 65, Sweden; Department of Ecology and Genetics/Animal Ecology, Uppsala University, Norbyvägen 18D, Uppsala 752 36, Sweden

**Keywords:** amphibians, *Bufotes viridis*, chomosome-level assembly, genome annotation

## Abstract

The European green toad (*Bufotes viridis*) is geographically widely distributed. While the species global conservation status is labeled as of least concern by the IUCN, it is declining in many parts of its range where populations are fragmented and isolated. A high-quality reference genome is an important resource for conservation genomic researchers who are trying to understand and interpret the genomic signals of population decline, inbreeding, and the accumulation of deleterious mutations. Here, we assembled and annotated a chromosome-level reference genome for *B. viridis* as part of the European Reference Genome Atlas pilot project. The genome assembly, with a size of ∼3.89 Gb consists of 11 chromosomes and an additional 2,096 unplaced scaffolds. The final assembly had a scaffold N50 value of 478.39 Mb and covered 90.4% single copy tetrapod orthologs, and 46.7% repetitive elements. Finally, a total of 23,830 protein-coding genes matching a known gene, together with 56,974 mRNAs were predicted. This high-quality reference genome will benefit amphibian evolutionary genomics research and enable conservation genetic studies to inform practical conservation work on this species.

## Introduction

Amphibians are considered to be the most threatened group of vertebrates, with more than 40% of the assessed amphibian species being threatened with extinction as a result of infectious disease, habitat loss, and climate change (Luedtke *et al*. 2023). Conservation genomics provides an approach for understanding basic biological properties relevant for the conservation of threatened taxa such as genetic diversity, population structure, amount of inbreeding, and genetic load (Formenti *et al*. 2022; Theissinger *et al*. 2023). As such, a high-quality reference genome is a valuable asset facilitating such work.

Among vertebrates, amphibians have the largest variance in genome size, ranging from 140 pg to as small as 0.95 pg (Liedtke *et al*. 2018). In anurans, the order consisting of frogs and toads, evidence suggests that the wide range in genome sizes is largely due to the recent accumulation of transposable elements, as well as the failure to remove ancient transposable element insertions (Zuo *et al*. 2023). Due to their genome size and complexity, amphibians have until fairly recently been largely overlooked by large scale initiatives to produce reference genomes, with only a small fraction of the ∼8,600 described amphibian species having a chromosome-level genome assembly available (Kosch et al. 2024).

The green toad genus *Bufotes* is geographically wide-spread and contains up of 15 recognized lineages. These include lineages that are diploid or allopolyploids (the latter having formed through hybrid speciation and that occurs to the eastern part of the *Bufotes* range) (Dufresnes *et al*. 2019). The *Bufotes viridis* subgroup occurs from Western Europe and the Northern part of the Mediterranean region to the Ural Mountains in the east, and is globally listed as of least concern but decreasing throughout its range (IUCN 2023). For example, in Sweden *B. viridis* has historically been declining and is considered to be the most vulnerable amphibian species in the country (Höglund *et al*. 2022), with its entire range confined to fragmented populations along the southern coast or on rocky islands off the coast, which are all geographically isolated from the European mainland populations.

Here, we present a chromosome-level, annotated reference genome for the pilot species *B. viridis* in the European Reference Genome Atlas (ERGA) pilot project (Mc Cartney *et al*. 2024). This reference genome will add to the growing number of amphibian reference genomes that will serve as useful tools for comparative and eco-evolutionary genomics studies, and to be used directly or indirectly to aid conservation practitioners working to safeguard amphibian diversity.

## Methods

### DNA and RNA extractions, sequencing library preparations, and sequencing

One male *B. viridis* from the ex situ breeding program at Nordens Ark (https://nordensark.se/bevarande/bevarande-i-sverige/gronflackig-padda/) was humanely euthanized and dissected by a veterinarian on the 2021 May 11 (Supplementary Fig. S1) with the approval from the Ethical committee for animal experiments in Göteborg (Dnr 5.8.18-06182/2021). HMW-DNA for PacBio Sequel II sequencing, frozen ground tissue for Omni-C and RNA for Illumina RNA-seq and PacBio Iso-Seq using 7 tissues (skin, spleen, breast muscle, lung, heart, liver, and kidney) were sequenced (See Supplementary Material online for details).

### Genome assembly

The PacBio HiFi reads were investigated for potential contamination using Mash Screen v2.3 (Ondov *et al*. 2019) against the NCBI RefSeq database (https://gembox.cbcb.umd.edu/mash/refseq.genomes.k21s1000.msh), and no contamination was detected. Further evaluation of coverage, genome size, and heterozygosity based on the HiFi reads was done using GeneScopeFK (https://github.com/thegenemyers/GENESCOPE.FK) using a k-mer size of 31, which is a modified version of GenomeScope v2.0 (Ranallo-Benavidez *et al*. 2020).

The PacBio HiFi https://www.doi.org/10.5281/zenodo.3552717 reads were assembled using IPA v.1.5.0 (https://github.com/PacificBiosciences/pbipa). The initial assembly, the purged assembly and the scaffolded assembly were evaluated with BUSCO v5.4.6 (Manni *et al*. 2021) and K-mer frequency histogram and completeness statistics produced using MERQURY.FK (https://github.com/thegenemyers/MERQURY.FK), as well as general assembly summary statistics using QUAST v.5.0.2 (Gurevich *et al*. 2013) (Table 1). The HiFi assembly was purged of duplicates using the IPA internal purge_dups v.1.2.5 (Guan *et al*. 2020). Omni-C reads were mapped to the contig assembly using BWA v. 0.7.17-r1188 (Li and Durbin 2009), with parameters −5SP and -T0. The alignments were then filtered and processed using pairtools v.0.3.0 (Open2C; Abdennur *et al*. 2023). The contig assembly was subsequently scaffolded using YaHS v.1.2.a.1.patch (Zhou et al. 2022). Manual curation of the scaffolded assembly was performed in Juicebox v.2.16.00 (Durand *et al*. 2016), and finalized using the juicer post command in YaHS.

**Table 1.** Summary statistics for the final assembly, gene, and repeat annotation.

Assembly statistics	
Contig L50	168
Contig N50 (Mb)	6.73
Longest contig (Mb)	37
Scaffold L50	3
Scaffold N50 (Mb)	478.39
Longest scaffold (Mb)	643.6
Number of scaffolds	2,107
Annotation statistics	
Number of protein-coding genes	31,608
Number of mRNA	69,426
Number of single exon genes	7,746
Average number of exons per mRNA	7.9
Average exon length	310
Average intron length	4,984
Repeat statistics	
DNA_transposons	366,392
LINE	178,322
LTR	110,821
LOW_complexity	183,696
RC	1,348
Retroposon	41
SINE	2,398
Satellite	3,213
Simple_repeat	1,016,805
Unknown	2,005,319
rRNA	1,845
scRNA	1
snRNA	18,749
srpRNA	1
tRNA	949

### Genome annotation

Using the Nextflow pipeline AnnotationPreprocessing (https://github.com/NBISweden/pipelines-nextflow), an initial annotation preprocessing step was performed by assessing assembly statistics, presence of missing and ambiguous nucleotides, gene completeness, presence of organelles, and distance to other annotated species.

Curated protein sequences were downloaded from the UniProt database (Magrane and UniProt Consortium 2011, downloaded on 2022-12; 568,363 proteins), including the amphibian UniProt database (38,940 proteins, uniprot_amphibia_PE12.fasta). A species specific repeat library was created using RepeatModeler v.2.0.2a (Smit, Hubley and Green 2010; Flynn *et al*. 2020) and RepeatRunner (Yandell 2006). The identified repeats (excluding transposons), were then matched against the collected protein set in order to exclude nucleotide motifs originating from low-complexity coding sequences. Repetitive sequences were then masked in the genome using the identified repeats with RepeatMasker v.4.1.2_p1 (Smit and Hubley 2010). Illumina RNA-seq data were aligned and assembled using an in-house pipeline from National Bioinformatics Infrastructure Sweden (NBIS) (https://github.com/NBISweden/pipelines-nextflow/tree/master/subworkflows/transcript_assembly) that includes fastq preprocessing in fastp v. 0.23.2 (Chen *et al*. 2018), alignment of reads to the rehttps://www.doi.org/10.5281/zenodo.3552717ference genome using HISAT v. 2.1.0 (Kim *et al*. 2015) and transcript assembly using StringTie v. 2.2.1 (Pertea *et al*. 2015).

The final assembly was annotated using the MAKER pipeline v.3.01.02 (Holt and Yandell 2011). First, evidence-based-annotations were constructed using both Illumina RNA-seq and Iso-seq assembled transcripts and Uni-Prot reference proteins. Second, using the initial MAKER evidence file, ab initio training was performed with Augustus v.3.3.3 (Stanke *et al*. 2006) using an NBIS in-house pipeline (https://github.com/NBISweden/pipelines-nextflow/tree/master/subworkflows/abinitio_training).

After running MAKER on a combination of evidence annotation together with curated ab initio profiles, a statistical evaluation of the final annotation was performed using the script agat_sp_statistics.pl from the AGAT package v.0.8.0 (Dainat 2021) followed by complimenting the final evidence-based annotation with the ab initio genes missing in the evidence-based build with agat_sp_complement_annotations.pl from the same toolkit.

Using BLAST v. 2.9.0 (Altschul *et al*. 1990), searching against the Uniprot/Swissprot databases, with a maximum e-value cutoff of 1e-6, and Interproscan v. 5.59–91.0 (Hunter *et al*. 2012) functional annotation was performed on the translated CDS features of each coding transcript using the NBIS in house pipeline functional_annotation (https://github.com/NBISweden/pipelines-nextflow/tree/master/subworkflows/functional_annotation). Furthermore, tRNAs were predicted using tRNAscan-SE v.1.3.1 (Lowe and Eddy 1997) and other ncRNAs were predicted using curated Rfam co-variance models (Nawrocki *et al*. 2014) with the infernal package v.1.1.2 (Nawrocki and Eddy 2013).

## Results and discussion

From an adult male *B. viridis* (Supplementary Fig. S1), we generated 149.53 Gb PacBio HiFi, 468.78 Gb Omni-C and 12.65 Gb Iso-Seq sequencing reads together with 32.56 Gb Illumina RNA-seq data from 7 tissues (Supplementary Table S1). Using Genomescope2 with a k-mer length of 31, the predicted genome size was ∼3.9 Gb with 0.27% heterozygosity (Supplementary Fig. S2). After purging of the initial HiFi assembly (Supplementary Fig. S3 and Table S2), we obtained a completeness score of 99.97 and QV score of 52.6 from MERQURY.FK (Supplementary Table S3). The final scaffolded assembly had a total size of ∼3.89 Gb and consists of 2,107 scaffolds (Supplementary Table S4). The longest scaffold is ∼644 Mb and the assembly has a scaffold N50 of ∼478 Mb (Table 1), with 43% GC content (Supplementary Table S2). Eleven of the 2,107 scaffolds were considerably larger than the others (Supplementary Fig. S4) suggesting that they correspond to the 11 chromosomes. This is in accordance with other published true toad genome assemblies (*Bufo gargarizans*: Lu *et al*. 2021, *Bufo bufo*: Streicher *et al*. 2021).

BUSCO scores using the tetrapoda_odb10 dataset containing 5,310 genes, for the final scaffolded assembly results in 91.3% complete tetrapod BUSCOs, out of which 90.4% are complete single copy and 0.9% complete duplicated BUSCOs (Supplementary Table S5).

A total of 46.7% (∼1.81 Gb) of the genome was repeat masked with the combined repeat annotations from RepeatModeler v.2.0.2a (Smit and Hubley 2010; Flynn *et al*. 2020) and RepeatRunner (Yandell 2006) (Supplementary Table S7). The majority of the repeats were classified as unknownl the rest consisted mostly of simple repeats, DNA transposons, LINEs, and LTRs (Table 1). This distribution of repeat families agrees with the expected distribution in Anuran genomes (Zuo *et al*. 2023).

**Fig. 1.**
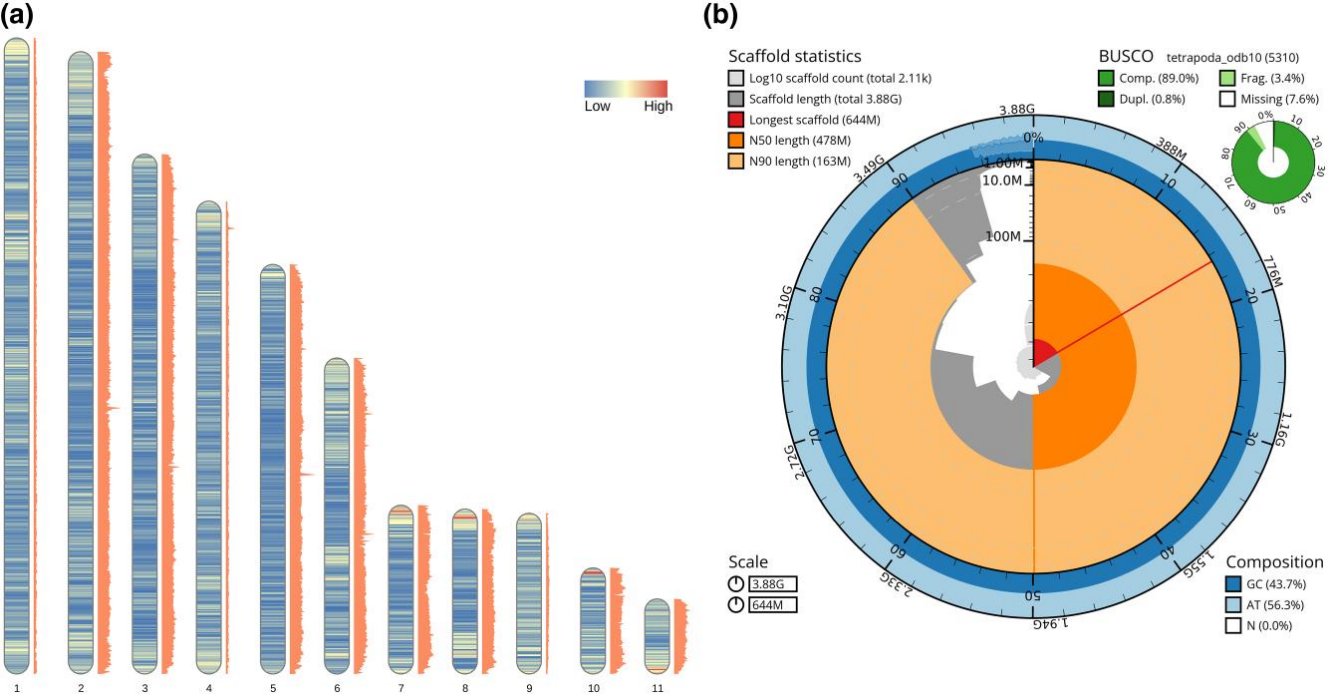
a) The 11 putative chromosomes order by size with gene density represented as an overlaid heatmap and density of repeats in orange on the right side of each chromosome RIdeogram v.0.2.2 (Hao *et al*. 2020). The largest chromosomes have a total length of 644 Mb. b) snail plot produced in BlobToolKit (Challis *et al*. 2020) showing assembly statistics and BUSCO scores for the final scaffolded assembly.

The initial run of the MAKER software failed for scaffolds (chromosomes) 1, 4, and 11 using the species-specific repeat library for the toad. For those chromosomes, the built-in repeat library in MAKER was instead used in separate runs, and the 2 runs with the species-specific and default repeat library were then merged. The relative repeat densities across chromosomes 1, 4, and 11 suggest that the reason behind this might be that the species-specific repeat identification and masking for those chromosomes might not have been entirely optimal together with the large size of the chromosomes (Fig. 1a).

The structure of a total of 31,608 protein-coding genes was annotated, with an average exon and intron length of 310 and 4,984 bp, respectively (Table 1). The annotated gene set covers ∼87% of complete tetrapod BUSCOs (Supplementary Table S6). Due to ab initio predictions showing elevated numbers of duplicated genes and fewer mRNAs, this final annotation was built based on evidence facilitated by Illumina RNA-seq and 219,633 full length cDNAs from Iso-Seq and complemented with ab initio predictions for loci that was missing in the initial evidence-based annotation. Furthermore, an elevated number of single exon genes without annotation was found and subsequently removed in the final build. In total 31,356 genes and 68,100 mRNAs could be linked to functional and protein domains (Supplementary Table S8), out of which 23,830 genes and 56,974 mRNAs could be assigned a name (e-value < 1e-6) by BLAST searches against the Uniprot/Swissprot database. Furthermore, a total of 5,922 tRNAs and 6,112 exons were predicted by tRNAscan-SE v.1.3.1 (Lowe and Eddy 1997) with a total exon length of 434 Kb. However, only 23 tRNAs remained after discarding annotations with an annotation edit distance of 1. The ∼66 Kb candidate sex determination locus for male-heterogametic XY found in Kuhl *et al*. 2024, consisting of the gene bod1 l (biorientation of chromosomes in cell division 1 was annotated and located in the region chr1: 579239124-579305844.

Finally, comparing the *B. viridis* assembly to the assemblies of the common toad (*Bufo viridis*, NCBI: GCF_905171765.1) and the Asiatic toad (*B. gargarizans*: GCF_014858855.1) reveal that while the genome size for *B. viridis* is smaller than the other 2 species, the contiguity (scaffold N50, Supplementary Table S9) is lower compared to both other assemblies but comparable to the Asiatic toad assembly. This is a result of the final assembly containing a relatively large number of unplaced scaffolds, the majority of which (67%) are under 100 Kb. It should also be noted that the 2 *Bufo* assemblies utilizes additional data for scaffolding (10X Genomics Chromium and BioNano data).

## Supplementary Material

jkaf002_Supplementary_Data

## Data Availability

The final assembly and raw data have been deposited at the European Nucleotide Archive under the project accession number: PRJEB71764. Files are also available via the ERGA portal (https://portal.erga-biodiversity.eu/data_portal/Bufotes%20viridis). Supplemental material available at G3 online.
