# Supplementary material for: A chromosome-level genome assembly of the European green toad (Bufotes viridis)
Source: G3 (Bethesda). 2025 Feb 19;15(3):jkaf002. doi: 10.1093/g3journal/jkaf002 (PMC11917475; doi:10.1093/g3journal/jkaf002)
Supplement: jkaf002_Supplementary_Data [file jkaf002_supplementary_data.pdf]

Supplementary information

Methods

HMW-DNA was extracted from 25 µl snap frozen blood collected from the body cavity, using the Monarch HMW DNA Extraction Kit for Cells & Blood (NEB) following the protocol "High Molecular Weight DNA Extraction for frozen nucleated blood" described in "Monarch HMW DNA Extraction Kit for Cells & Blood Version 1.0_10/20". DNA was eluted in 600 µl kit EB elution buffer and stored at 4° C.

70 ug of DNA was used for Shearing on the Megaruptor® 2 DNA Shearing System to ~20kb followed by SMRTbell library construction. Primer annealing and polymerase binding were performed using the Sequel II binding kit 2.2 and Sequencing Primer v5. Size selection was performed using the SageELF system (SageScience). Finally, six Sequel™ SMRT® Cells 8M v3 (Sequel SMRT Cells were supplied by PacBio, Menlo Park, US) were sequenced on Sequel II and IIe system using Sequel® II Sequencing Plate 2.0, On-Plate Loading Concentration of 80 pM, movie time 30 hours and pre-extension time 2 hours. Since the strategy was to sequence the longest reads possible to get the most contiguous assembly, the majority of mitochondrial reads were excluded during size selection, and we were therefore unable to assemble the mitochondrial genome.

Using the Dovetail Omni-C kit, liver samples were processed using the non-mammalian samples protocol v1.2 Method B. A total of 26 mg of frozen, ground tissue were defrosted and crosslinked for 10 minutes in 3 mM disuccinimidyl glutarate (DSG) followed by an additional crosslinking of 10 minutes in 1 % Formaldehyde. 1ml syringe and 200 µM/ 50 µM filter were used to remove debris from tissue. The digestion conditions for the chromatin were determined by titration of the kit-provided Nuclease Enzyme Mix. Two reactions of proximity ligation consisting of end-polishing, bridge ligation, intra-aggregate ligation and crosslink reversal were performed as

described by the supplier using the Omni-C Kit (Dovetail, DG-REF-001, DG-REF-002) and 1000 ng input per reaction. The chromatin was purified using AMPure XP beads (Beckman Coulter, A63882).

A total of 67 ng and 127 ng of each proximity-ligated chromatin reaction was used as input for preparation of one library each using reagents from NEBNext® Ultra™ II DNA Library prep kit for Illumina (NEB, E745S). The indexing PCR step used NEBNext® Multiplex Oligos for Illumina® Index Primers Set 1 (NEB, E7335S) and indexed libraries were subjected to 12 amplification cycles prior to DNA cleanup using AMPure XP beads. The final libraries were analyzed for fragment length and concentration using a Bioanalyzer DNA high sensitivity chip and Qubit high sensitivity dsDNA. Libraries were then pooled and sequenced on an Illumina NovaSeq6000 (NovaSeq Control Software 1.7.5/RTA v3.4.4) with a 151nt(Read1)-10nt(Index1)-10nt(Index2)-151nt(Read2) setup using the 'NovaSeqXp' workflow in 'S4' mode flowcell.

In total, seven tissues (skin, spleen, breast muscle, lung, heart, liver and kidney) were sequenced with Illumina RNA-seq and PacBio Iso-Seq to be used for genome annotation. Individual Illumina libraries were prepared for each tissue, and for PacBio Iso-Seq equimolar amounts of RNA were pooled into one sample before the library preparation step. Total RNA was extracted from the seven tissues using a standard TRIzol protocol including DNase treatment (RNeasy, Invitrogen). RNA was extracted from cryo-ground tissues using the TRIzol Reagent and Phasemaker Tubes Complete System (Invitrogen Cat #A33250) following the Invitrogen user guide (Pub. No. MAN0016163 Rev. A.0) except for steps 2.a. and 3.d., which were omitted. RNA was re-suspended in 87.5 µl RNase-free water and immediately subjected to DNase treatment followed by purification according to the RNeasy Micro Handbook (pages 74 and 53). The eluted RNA was stored at -70° C until processed further.

Illumina RNA-seq libraries were prepared using a polyA capture protocol with the Illumina Stranded mRNA Prep kit, following the manufacturer's instructions and using MagBio HighPrep PCR magnetic beads for library cleanup. Library fragment size distribution and concentration were assessed using High Sensitivity D5000 ScreenTapes with a TapeStation 4150 (Agilent Technologies) and a Qubit Fluorometer (Thermo Fisher Scientific, Waltham, MA, USA), respectively. Libraries were denatured and loaded for paired-end sequencing on an Illumina NovaSeq 6000 system using a NovaSeq 6000 S1 Reagent Kit v1.5 (300 cycles). A dual-indexed running mode was set to 10:151:151:10 bp cycles. Illumina RNA-seq library preperation was performed at the ERGA node in Antwerp, Belgium and sequencing was performed at the ERGA node in Florence, Italy.

One IsoSeq SMRTbell library was prepared from equimolarly pooled RNA from multiple tissues as described in "Procedure & Checklist – Iso-Seq™ Express Template Preparation for Sequel® and Sequel II Systems" (PN 101-763-800 Version 02 (October 2019)) using the NEBNext® Single Cell/Low Input cDNA Synthesis & Amplification Module (New England Biolabs), the Iso-Seq Express Oligo Kit (PacBio), ProNex beads (Promega) and the SMRTbell Express Template Prep Kit 2.0 (PacBio). 300 ng of total RNA were used for cDNA Synthesis followed by 12 cycles of cDNA Amplification.

Based on the cDNA size estimation with Fragment Analyser (Agilent), the sample was primarily composed of transcripts around 2 kb in length. Hence, the standard workflow was applied for purification of cDNA and construction of Iso-Seq library, as described in manufacturer instructions.. After purification the amplified cDNA went into SMRTbell library construction. Primer annealing and polymerase binding was performed using the Sequel II binding kit 2.0 and Sequencing Primer v4. Finally, one Sequel™ SMRT® Cells 8M v3 was sequenced, one on Sequel IIe using Sequel® II Sequencing Plate 2.0, On-Plate Loading Concentration of 110 pM, movie time 24 hours and pre-extension time 2 hours. Wet-lab and sequencing of PacBio IsoSeq and HiFi was

performed at the the ERGA node SciLifeLab Genomics, Sweden. The sequenced male *B. viridis* is stored as a voucher specimen at the Evolution Museum at Uppsala University (Fig. S1) under accession number UPSZTY 184740.

Results

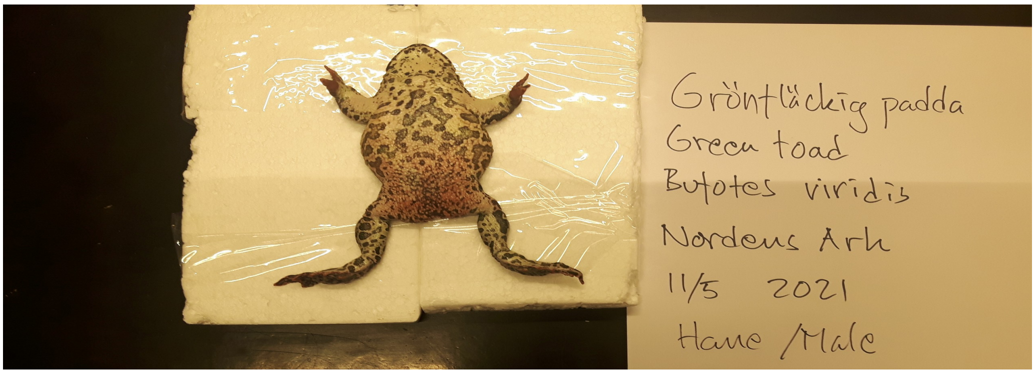

Fig. S1. Male green toad sacrificed for sequencing. Voucher number: UPSZTY 184740

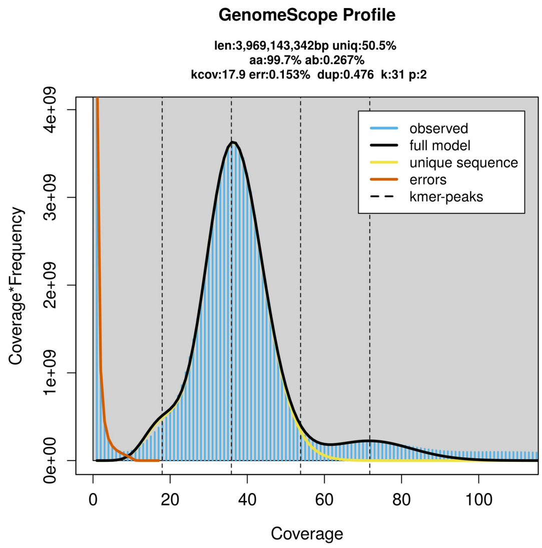

Fig. S2. Genomescope2 profile plot for k-mer 31 with associated summary statistics below the header.

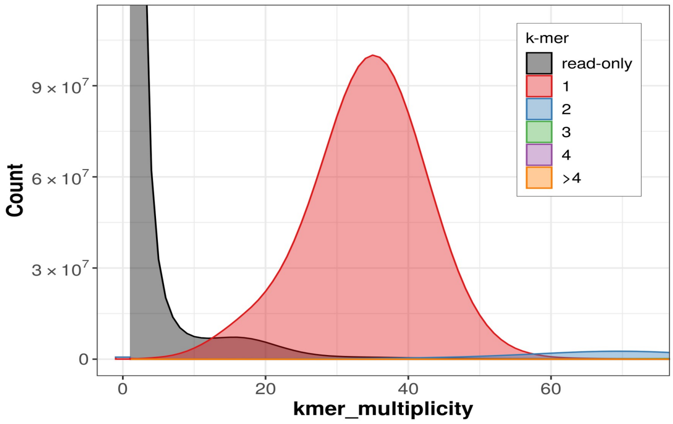

Fig. S3. Plot showing Merqury K-mer frequency spectrum plot for purged HiFi assembly.

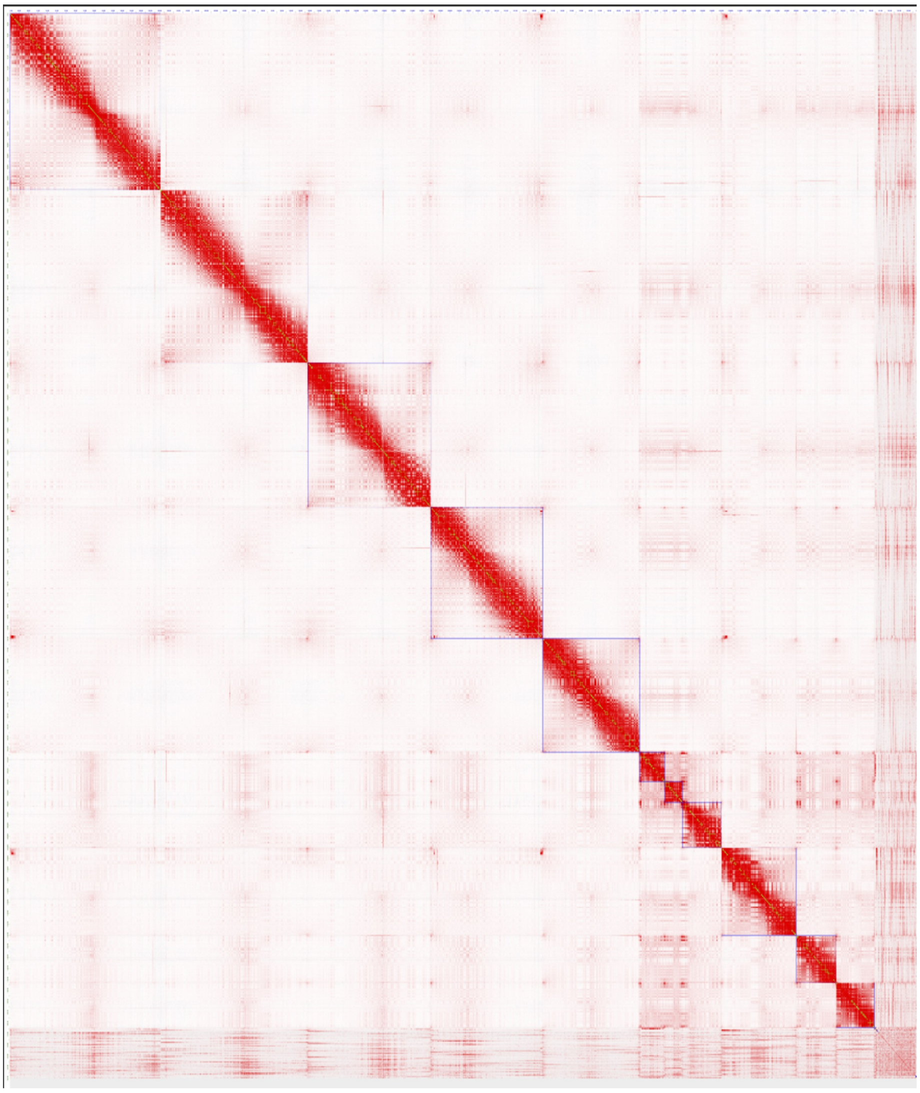

Fig. S4. Manually curated Hi-C contact map from Juicebox suggesting 11 chromosome like scaffolds.

Table. S1. Sequencing output.

Data	Output (reads/read-pairs)	Gb
PacBio HiFi	7,597,452	149.53
PacBio Isoseq (pool of 7 tissues)	3,564,332	12.65
Omni-c	3125.30M	468.78
Illumina RNA-seq (Heart)	39,354,917	5.91
Illumina RNA-seq (Kidney)	30,359,076	4.55
Illumina RNA-seq (Liver)	29,233,585	4.38
Illumina RNA-seq (Lung)	24,557,954	3.68
Illumina RNA-seq (Muscle)	28,244,752	4.24
Illumina RNA-seq (Spleen)	33,255,663	4.99
Illumina RNA-seq (Skin)	32,037,182	4.81

Table. S2. Quast assembly statistics for the purged IPA HiFi assembly.

HiFi assembly statistics (Quast)	
# contigs (≥ 0 bp)	3396
# contigs (≥ 1000 bp)	3396
# contigs (≥ 5000 bp)	3396
# contigs (≥ 10000 bp)	3390
# contigs (≥ 25000 bp)	3073
# contigs (≥ 50000 bp)	2302
Total length (≥ 0 bp)	3,879,462,136
Total length (≥ 1000 bp)	3,879,462,136
Total length (≥ 5000 bp)	3,879,462,136
Total length (≥ 10000 bp)	3,879,415,658
Total length (≥ 25000 bp)	3,872,725,642
Total length (≥ 50000 bp)	3,846,079,898
# contigs	3396
Largest contig	37,076,692
Total length	3,879,462,136
GC (%)	43.68
N50	6,828,217
N75	2,915,537
L50	166
L75	382
# N's per 100 kbp	0.05

Table. S3. Merqury completeness, QV and false duplication statistics on purged IPA HiFi assembly

**MerquryFK completeness**	**MerquryFK QV**	**Merqury false duplication**
99.97	52.6	0.6

Table. S4. Quast assembly statistics for the final assembly.

Final assembly statistics (Quast)	
#contigs (≥ 0 bp)	2,107
#contigs (≥ 1000 bp)	2,107
#contigs (≥ 5000 bp)	2,098
#contigs (≥ 10000 bp)	2,092
#contigs (≥ 25000 bp)	1,760
#contigs (≥ 50000 bp)	998
Total length (≥ 0 bp)	3,879,743,336
Total length (≥ 1000 bp)	3,879,743,336
Total length (≥ 5000 bp)	3,879,721,336
Total length (≥ 10000 bp)	3,879,674,858
Total length (≥ 25000 bp)	3,872,768,245
Total length (≥ 50000 bp)	3,846,471,649
#contigs	2,107
Largest contig	643,651,765
Total length	3,879,743,336
GC (%)	43.68
N50	478,304,964
N75	319,749,361
L50	4
L75	6
#N's per 100 kbp	7.30

Table. S5. BUSCO scores for the final assembly before annotation using tetrapoda_odb10.

BUSCO scores before annotation (tetrapoda_odb10)<br>C:91.3% [S:90.4%, D:0.9%], F:2.4%, M:6.3%, n:5310	
Complete BUSCOs (C)	4848
Complete and single-copy BUSCOs (S)	4799
Complete and duplicated BUSCOs (D)	49
Fragmented BUSCOs (F)	129
Missing BUSCOs (M)	333
Total BUSCO groups searched	5310

Table. S6. BUSCO scores for final the assembly after annotation on the predicted genes using tetrapoda_odb10.

BUSCO scores before annotation (tetrapoda_odb10)<br>C:86.7% [S:85.3%, D:1.4%], F:4.0%, M:9.3%, n:5310	
Complete BUSCOs (C)	4606
Complete and single-copy BUSCOs (S)	4530
Complete and duplicated BUSCOs (D)	76
Fragmented BUSCOs (F)	212
Missing BUSCOs (M)	492
Total BUSCO groups searched	5310

Table. S7. Repeat masking results from RepeatModeler and RepeatRunner.

Tool	Library	Number	Total size (kb)	Mean size (bp)	% Genome
RepeatMasker	De-novo	3,889,908	1,775,004.08	456.31	45.75
RepeatRunner	MAKER TE	50,839	36,265.82	713.35	0.93

Table. S8. Functional and protein domain annotation results from InterProScan.

Databases	Nb term linked to mRNA	Nb mRNA updated by term in our annotation file	Nb gene updated by term in our annotation file
CDD	33273	24096	10484
Coils	13566	13566	6713
FunFam	104655	35612	12682
Gene3D	81173	50440	20380
Hamap	1069	1015	396
InterPro	233155	61772	26744
MetaCyc	10648155	44858	18209
MobiDBLite	31457	31457	15665
Ontology_term	168722	61772	26744
PANTHER	61000	61000	26531
PIRSF	3206	3144	1552
PRINTS	23609	16853	5648
Pfam	88857	56432	23522
ProSitePatterns	27093	22388	6974
ProSiteProfiles	50899	36246	14104
Reactome	39710762	55389	22732
SFLD	650	321	107
SMART	44300	30264	11399
SUPERFAMILY	67113	47830	19172
TIGRFAM	3252	2934	1138

Table. S9. Comparison of assembly statistics from NCBI between the green toad (*Bufotes viridis*), Asiatic toad (*Bufo gargarizans*: GCF_014858855.1) and common toad (*Bufo bufo*: GCF_905171765.1).

Databases	Bufotes viridis	Bufo gargarizans: ASM1485885v1	Bufo bufo: aBufBuf1.1
Genome size	3.9 Gb	4.5 Gb	5 Gb
Total ungapped length	3.9 Gb	4.5 Gb	5 Gb
Number of scaffolds	2107	747	1306
Scaffold N50	478.3 Mb	539.8 Mb	635.7 Mb
Scaffold L50	4	4	4
Number of contigs	3599	4620	5501
Contig N50	6.7 Mb	1.7 Mb	4 Mb
Contig L50	169	749	350
GC percent	43.5	44.5	44.5
Genome coverage	30.0x	103.0x	64.0x
